# Supplementary material for: Sustainable biomimetic solar distillation with edge crystallization for passive salt collection and zero brine discharge
Source: Nat Commun. 2024 Jan 29;15:874. doi: 10.1038/s41467-024-45108-2 (PMC10825211; doi:10.1038/s41467-024-45108-2)
Supplement: Supplementary file 1 — Supplementary Information file [file 41467_2024_45108_MOESM1_ESM.pdf]

Supplementary Information

Sustainable Biomimetic Solar Distillation with Edge Crystallization for Passive Salt Collection and Zero-Brine Discharge

Mohamed A. Abdelsalam,[1+] Muhammad Sajjad,[1+] Aikifa Raza,[1] Faisal AlMarzooqi,[2] TieJun Zhang[1*]

[1]*Department of Mechanical and Nuclear Engineering,*

Khalifa University of Science and Technology, P.O. Box 127788, Abu Dhabi, United Arab Emirates

[2]*Department of Chemical and Petroleum Engineering,*

Khalifa University of Science and Technology, P.O. Box 127788, Abu Dhabi, United Arab Emirates

[+]These authors contributed equally; *Corresponding author: tiejun.zhang@ku.ac.ae

Supplementary Note 1: Fabrication and characterization of TiO_2/Ti mesh for solar vapor generator and crystallizer (SVGC)

Chemical oxidation:

The titanium mesh and the substrate of various sizes were oxidized in 1M aqueous sodium hydroxide solution using a hydrothermal approach. The hydrothermal reactor was heated to 220°C for 3, 15 and 25 hours. After the chemical oxidation, the mesh was rinsed three times using deionized water. The etching time affects the growth density of titanium dioxide nanostructures on wires of titanium mesh. The effective porosity of the titanium mesh is around 0.4.[1] Note that, the titanium samples were carefully cleaned by using ethanol and deionized water prior to oxidation process.

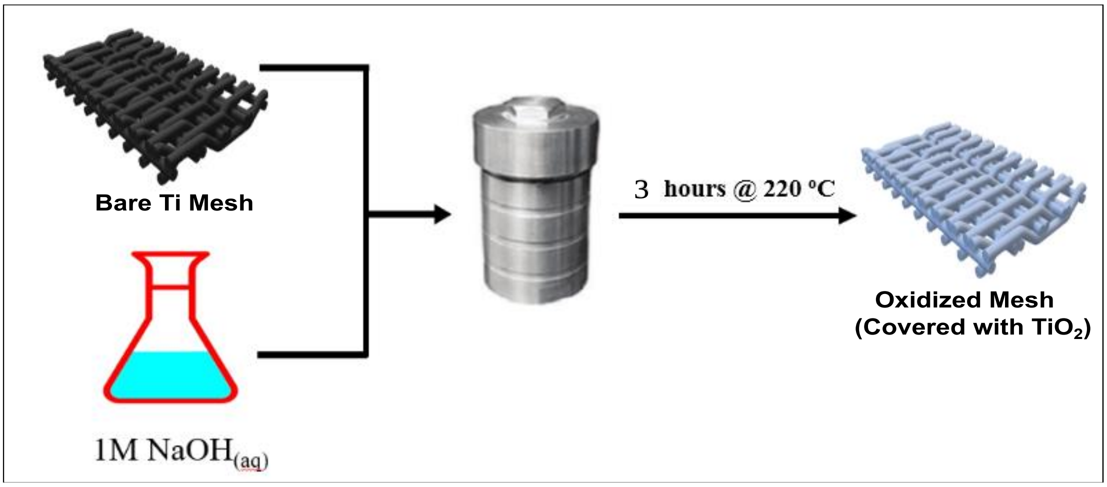

Supplementary Figure 1: Hydrothermal synthesis for the chemical treatment of titanium mesh and substrate.

Morphology: Figure S2 shows the optical images of bare titanium mesh before and after chemical oxidation for 3, 15 and 25 hours. The chemical oxidation time of titanium mesh is critical to control the growth of titanium dioxide nano/microstructures on mesh surface. After chemical oxidation for 3 hours, the color of titanium mesh surface changes from glossy greenish grey to nonglossy dark grey, while the longer oxidation time causes an obvious color change to bluish and whiteish grey. The former indicates the presence of lower concentration/growth of TiO_2 nanostructures, while the latter two cases show much higher concentration/growth of TiO_2 nanostructures at the mesh surface. This is confirmed by surface morphological characterization by using scanning electron microscopy (SEM), as shown in Fig. S2 (b-d). The SEM images show distinguishable TiO_2 nanostructures on both meshes, as

lower oxidation time results in submicron particle-like morphology, while the 15 h oxidation time results in dense grass-like TiO_2 nanowires with diameter below 100 nm. For prolonged oxidation time of 25 h, the grass-like TiO_2 nanowires started to diffuse, showing cluster-like morphology.

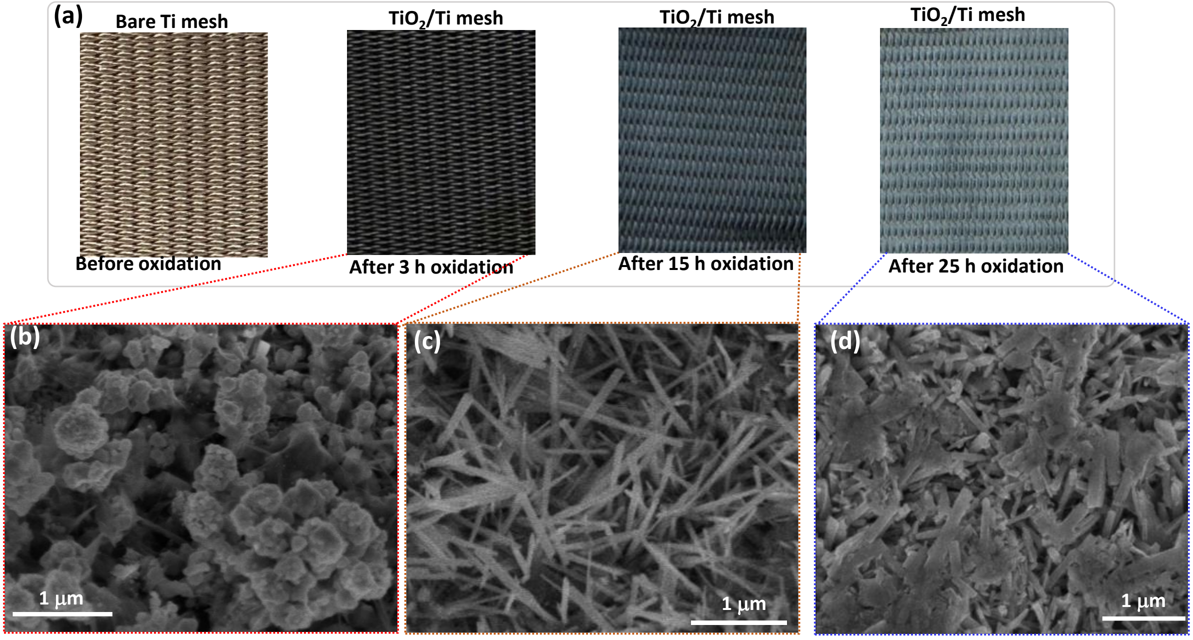

Supplementary Figure 2: (a) Optical images of titanium mesh before and after chemical oxidation for 3, 15, and 25 hours. (b-d) Scanning electron microscopic (SEM) images of respective oxidized TiO_2/Ti mesh.

XRD characterization: In addition, we also characterized the crystallinity and phases of TiO_2/Ti mesh using X-ray diffraction (XRD) technique (PANalytical Empyrean). Figure S3 (a-b) shows the XRD pattern of titanium mesh before and after oxidation which indicates the presence of diffraction peaks corresponding to the TiO2 nanostructures and Ti substrate. The typical diffraction peak (101) centred at 24.9° indicates the presence of dominant TiO2 anatase phase. Except for the peaks corresponding to metal titanium, the diffraction peaks of TiO2/Ti match well the TiO2 anatase phase peaks with traces of rutile phase,, as shown in Fig. S3 (b).[2,3] The strongest peaks at $2\theta = 24.9°$, 48.0°, and 53.9° are corresponding to the (101), (200) and (105) crystalline planes of anatase phase, while the weak peaks at $2\theta = 61.3°$ and 63.5° represent the (002), (310) crystalline planes of rutile phase, respectively.

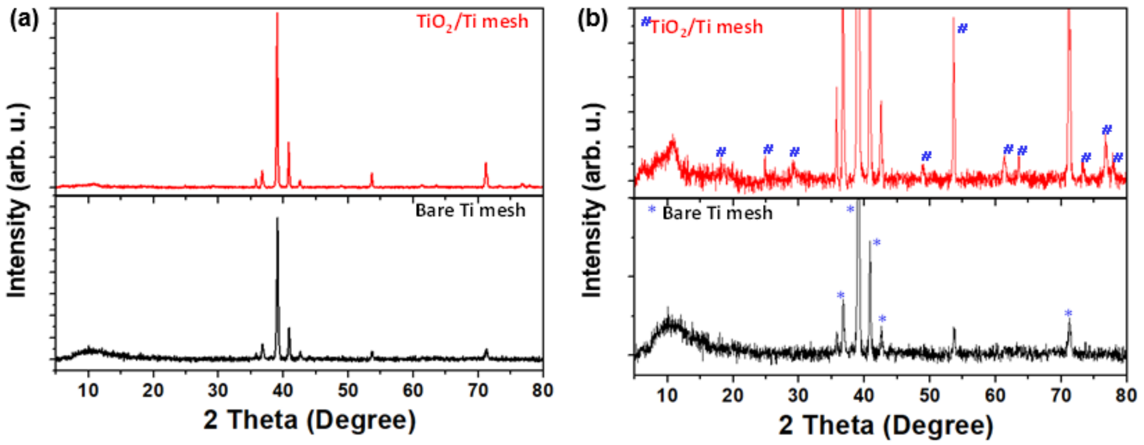

Supplementary Figure 3: (a) XRD spectra of titanium mesh before and after oxidation. (b) zoom in of spectra (a) to show the diffraction peaks with lower intensities.

Optical characterization: It is worth mentioning that both the effective light absorption and superhydrophilicity for continuous water supply are essential for high-performance SVGC devices. Moreover, we also performed the optical (Perkin Elmer, Lambda 1050) and wettability (Kyowa, contact angle goniometer) characterization. Figure S4 shows the measured absorption spectra of various TiO_2/Ti meshes in the wavelength range of 250-2000 nm. TiO_2/Ti meshes after oxidizing for 3 h exhibit higher light absorption capability than those after oxidizing for 25 h, because TiO_2 nanostructure in the latter case has the higher reflectivity.

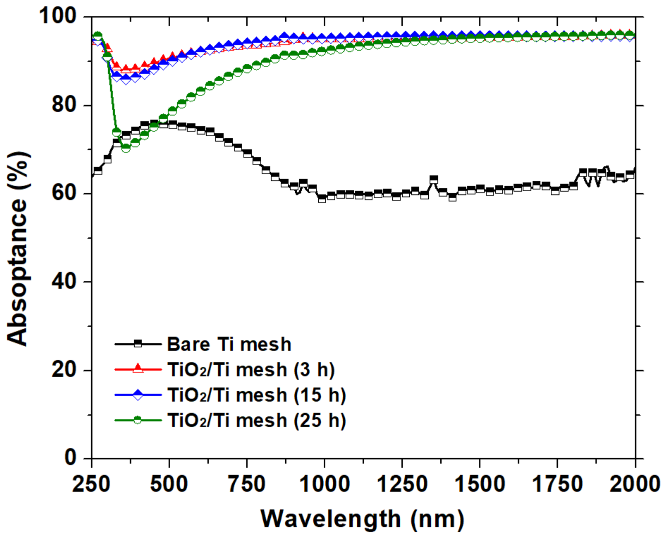

Supplementary Figure 4: Comparison of measured absorption spectra of as-prepared TiO_2/Ti mesh after oxidizing for 3, 15, and 25 h in the wavelength range of 250-2000 nm. The red and black curves are reproduced with permission from [1].

Wettability characterization: For wettability characterization, we have observed the water droplet spreading behavior on mesh and substrate via high-speed optical imaging. Figure S5 shows the optical images of water droplets in contact with bare titanium and TiO_2/Ti substrates. Bare titanium shows the water contact angle of 66±2.2° after 425 ms, while TiO_2/Ti substrate shows the superhydrophilicity with a water contact angle of 6.3±0.5°. Time-lapse optical snapshots of water droplets spreading on bare titanium and TiO_2/Ti meshes are shown in Fig. S6. Water droplets did not wet the bare titanium mesh while instantly spreading on nano/microstructured TiO_2/Ti mesh. Notably, we did not observe any leakage of water across the TiO_2/Ti meshes after 150 ms as shown in Fig. S6.

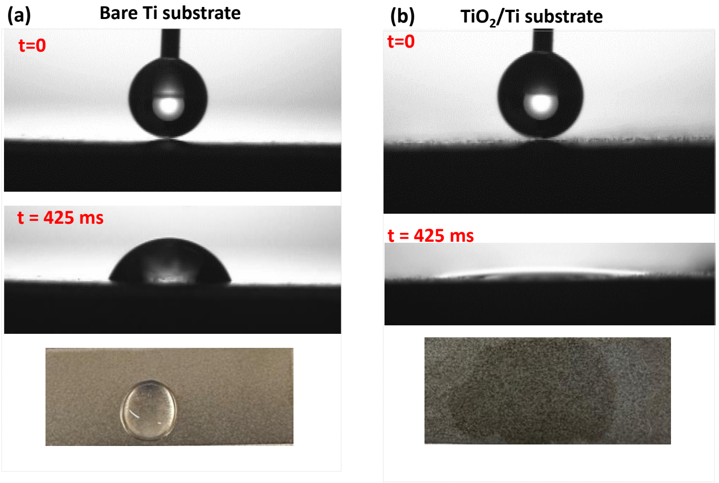

Supplementary Figure 5: Optical images of water droplets in contact with (a) bare titanium and (b) TiO_2/Ti substrates.

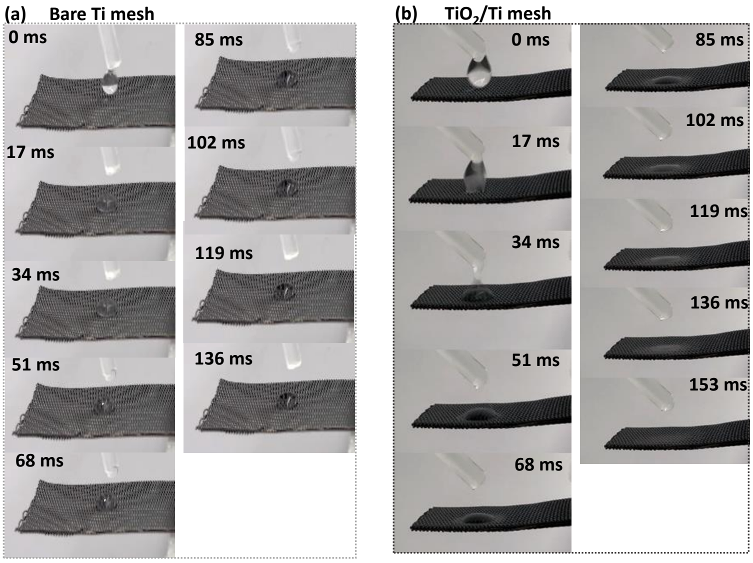

Supplementary Figure 6: Time lapse image showing water droplet spreading on (a) bare titanium and (b) oxidized TiO_2/Ti mesh.

Foldability of mesh: Our foldable biomimetic solar evaporator is made of chemically etched titanium meshes. As shown in Fig. S7(a) of supporting information, it can retain its shape when folded and bent without fracture. Therefore, we cut and folded the mesh to attain desired shapes such as small "tree" with four leaves and a big "tree" with many branches at different heights, as shown in Fig. S7(b-c). Mass change comparison for two kinds of devices show the impact of increasing the number of evaporative leaves (Fig. S7(d)). Besides tree-shape SVGC structure, the mesh was also bent at various title angles to fabricate single-leaf solar vapor generator for studying the effect of tilt angle on evaporation and salt crystallization. Evaporating area changes with the bending angle under the same projection area for light illumination. Owing to the foldable nature of mesh, the leaves of tree-like SVGC device are oriented/folded for maximum exposure to the sunlight during outdoor vapor generation experiments. Moreover, our SVGC device can be used as portable freshwater generator in remote areas while saving space compared to bulky devices.

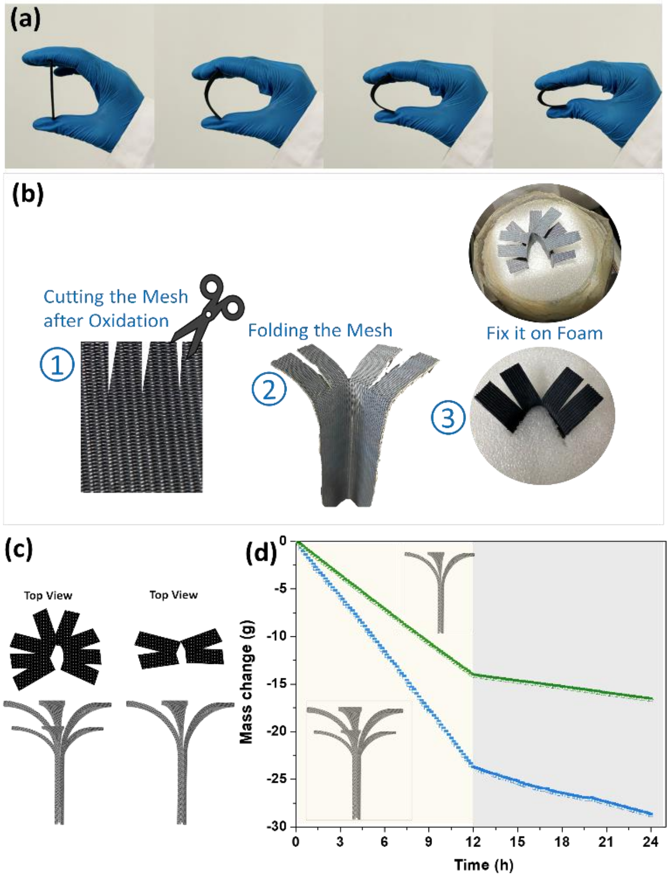

Supplementary Figure 7: (a) Optical images showing the foldability of titanium mesh used as SVGC. (b) Schematic illustration showing mesh cutting and folding to form flower or tree like device. (c) Schematic showing top and side view of tree like structure with different stem height and number of leaves (d) Mass change comparison for two tree like devices.

Mesh geometry: The structure of the titanium mesh used in SVGC device is similar to the twilled dutch type mesh. There are three shute wires in a repeating pattern and eight warp wires in pairs in a representative unit cell (Fig. S8). 3D model of the mesh prepared in Solid Edge is shown in Fig. S8a. Similarly, Fig. S8b shows the 2D view of the lateral (shute wires weaving direction) cross-section of the unit cell while Fig. S7c shows the 2D view of the longitudinal (perpendicular to warp wires direction) cross-section of the unit cell. The mesh wires are tightly weaved to each other, and no large pore is observed. Both shute and warp wires have the same diameter of 0.27 mm. The warp wire pitch is 1.33 mm while the mesh thickness is 0.81 mm.

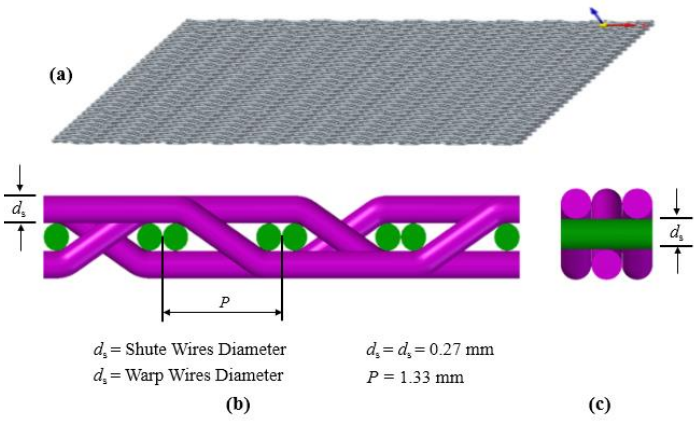

Supplementary Figure 8: (a) 3D model of the titanium mesh, 2D view of the (b) longitudinal and (c) lateral cross-sections of mesh unit cell. Wires in violet color are warp wires while in green color are shute wires.

Supplementary Note 2: Evaporation Flux and Thermal Efficiency Calculations

The evaporation flux (evaporation rate) was calculated under steady-state conditions through normalizing the mass loss obtained from the electronic balance by the total area of the solar evaporator (top + bottom + sides) and solar illumination time. The evaporation flux was calculated for the dark evaporation and under solar irradiance in indoor lab conditions. The evaporation flux can be expressed as:

$$Evaporation\ Flux = \frac{\Delta m}{A_{total}\Delta t} \quad \text{(S1)}$$

The solar vapor generation performance can be evaluated by determining the evaporation efficiency. The light to heat conversion efficiency can be defined as the ratio of enthalpy difference in the produced vapor (total enthalpy of phase change during the conversion of seawater from liquid to steam) over the total incoming solar irradiance as expressed in Eq. S2.

$$\eta_{th} = \frac{\dot{m}(h_{fg}+C_p\Delta T)}{\alpha q_s A} \quad \text{(S2)}$$

Where $\dot{m}$ represents the transient change in mass during the evaporation process in kg/s, h_{fg} is the enthalpy change from liquid to water vapor $\approx 2400\frac{kj}{kg}$, α is the average light absorption q_{solar} is representing the incident solar flux per area W/m^2, and A is the solar exposed area of the absorber (top illuminated area in our scenario). The energy required for vaporization is divided into two parts, the energy associated with the phase change from liquid to vapor i.e. $\dot{m}h_{fg}$. The symbol $\dot{m}$ is the evaporation rate in kg/s and h_{fg} is the latent heat of vaporization for the saline water at given salinity and temperature. The second part $\dot{m}C_p\Delta T$ is the sensible heat, and C_p is the specific heat of the saline water. It is noteworthy that the sensible heat is much lower than the latent heat. For instance, for pure water, sensible heat for the temperature gradient of 10 ^{o}C between the absorber and bulk water (as in the case of our device) is estimated to be 42 kJ/kg while latent heat of vaporization at 35 ^{o}C is h_{fg} = ~ 2422 kJ/kg. Therefore, the sensible heat is less than 1% of the total useful heat and can be neglected. Evaporation efficiency could be employed as a crucial tool to compare several solar interfacial desalination devices globally.

Supplementary Note 3: Heat Loss Analysis

Under normal operating conditions, heat loss through radiation, convection, and conduction from the absorber to bulk water will also occur. **Fig S9** shows the schematic representation of the energy balance of the proposed flower-shaped evaporation device.

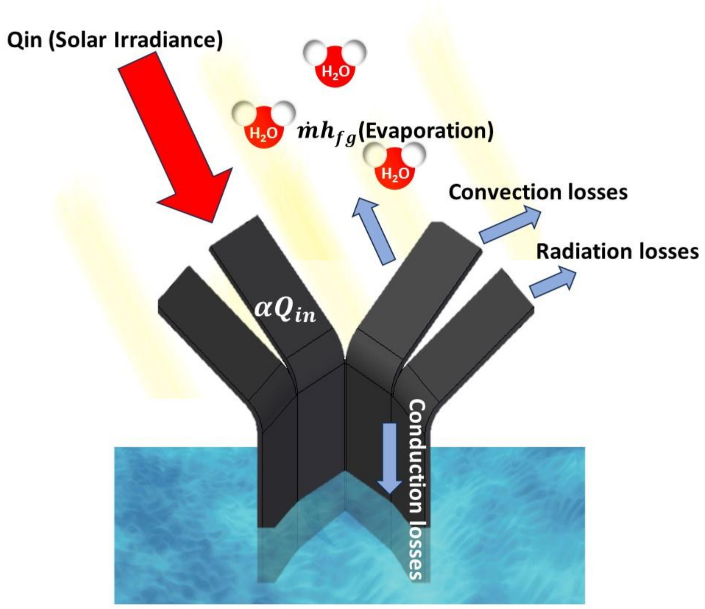

Supplementary Figure 9: Schematic diagram of the energy balance of the system

The energy balance for our SVGC device can be written as:

$$\dot{q}_{sol} = \dot{m}_{loss}\left(h_{l-v} + \dot{c}_p\, \Delta T\right) + \dot{q}_{cond} + \dot{q}_{rad} + \dot{q}_{conv} \qquad \text{(S3)}$$

$\dot{q}_{sol}$ is solar energy which is product of illuminated area, average absorptivity and received solar irradiance (or flux). The conduction heat loss ($\dot{q}_{cond}$) happens from SVGC to the bulk solution and can evaluated as:

$$\dot{q}_{cond} = k_{eff} A_c \frac{(T_{SVGC} - T_{\infty})}{h_{SVGC}} \qquad \text{(S4)}$$

where k_{eff} is the effective thermal conductivity of the mesh in W/m^2 K, A_c is the cross-sectional area in m^2 and h_{mesh} is the distance from SVGC surface to bulk solution in m.

The radiative energy transfer from SVGC to ambient can be evaluated as:

$$\dot{q}_{rad} = \sigma_{SB} \varepsilon_{SVGC}\, A_s (T_{SVGC}^4 - T_{\infty}^4) \qquad \text{(S5)}$$

Where A_s represents the total surface area of the mesh in m^2, ε_{SVGC} is the emissivity of the mesh and σ_{SB} is the Stefan-Boltzmann constant. The convection heat loss (q_{conv}) takes place from SVGC to ambient air expressed as:

$$\dot{q}_{conv} = h_{conv} A_s (T_{SVGC} - T_{\infty}) \qquad \text{(S6)}$$

where h_{conv} represents the heat transfer coefficient (~ 5 W/m^2).

Supplementary Note 4: SVGC Device Performance

Effect of tilt angle on single leaf evaporator: As shown in the respective IR images in Fig. 2b main text, the steady-state temperature of the water in the first case was 26 ℃ and the evaporation flux was as low as 0.06 kg $m^{-2}h^{-1}$ (Fig.S10) due to the lack of heat localization and a relatively big area of the liquid/air interface. For the negatively tilted leaf of SVGC, the evaporation flux was the highest (0.78 kg $m^{-2}h^{-1}$), when compared to others at the first hour of illumination. However, with the continuous evaporation of saline water and owing to the strong liquid pumping and gravity effect, a visible crusty salt layer tended to progressively accumulate on the single leaf SVGC (optical images in Fig. 2b main manuscript and Fig. S10). This would hinder light absorption of the leaf, subsequently increase the surface temperature to 38℃ and imperatively deteriorate the generated vapor to escape, thus reducing the light-to-heat conversion efficiency of the solar vapor generator and the evaporation flux. Moreover, crystalizing the salt on the top of the leaf results in back diffusion of the salt to the bulk water container when switching off the light source, which would eventually increase the salinity of the bulk instead of harvesting the salt without discharge. Similarly, the straight horizontal evaporator started with a high evaporation flux of 0.7 kg $m^{-2}h^{-1}$, then its flux decreased to 0.55 kg $m^{-2}h^{-1}$ (red curve in Fig 2c main manuscript) because of the rapid salt crystallization on the leaf surface, driven by uniform heating from the solar simulator on the top leaf surface. The crusty salt crystals inevitably increased the light reflection under direct light irradiance, resulting in a sharp decrease in evaporation flux and thermal efficiency over time. The temperature of the top horizontal surface was 33℃, as shown by the IR images in Fig. 2b main text. In contrast, for the positively tilted leaf, the evaporation flux was initially reduced to 0.65 kg m^{-2} h^{-1} from 0.77 kg $m^{-2}h^{-1}$ and then increased to be stable at about 0.7 kg m^{-2} h^{-1} (blue curve in Fig. 2c main manuscript), since the evaporation surface was eventually clean without the salt accumulation and with a relatively low temperature of 30 ℃, as shown by the optical and IR images in Fig. 2b main manuscript. It was noticed that after 12 hours of illumination, a thick salt layer was accumulated on the edges of the leaf. Edge-preferred crystallization is beneficial for salt harvesting since the continuous accumulation of salt on the edge will eventually fall off by the action of gravity, maintaining a salt-free evaporation surface for continuous vapor generation and mineral recovery from seawater.

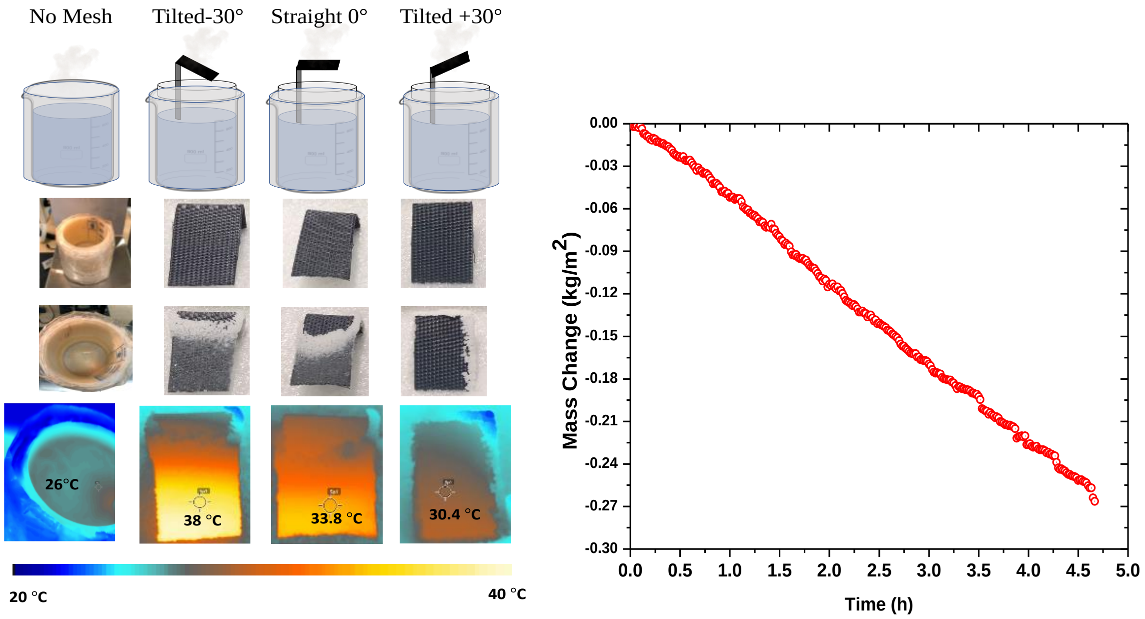

Supplementary Figure 10**:** Left image: optical and IR images of four various setups using saline water with salinity of 12wt% under one sun illumination: with no mesh, single leaf solar vapor generator with tilt angles of -30, 0 and +30°. Mass Change for water without evaporator under 1 sun irradiance.

Comparison between short and long evaporator: As presented by the IR images in Fig. 2e (main manuscript), after 12 hours of continuous illumination, the long stem in mangrove-like SVGC with 3 cm height (above the top insulating foam) recorded a temperature of 26 ℃ on the top leaf surface and 22 ℃ on the stem, while the short stem has the relatively high surface temperature of ~30 ℃ (IR images in Fig 2e main manuscript). This is because the large evaporation area of long stem increases the total water mass loss with the contribution from dark evaporation. The absolute water mass loss from the long stem in mangrove- like SVGC recorded a cumulative value of 28 g when compared with the 18 g for the short stem. However, when the mass loss values were normalized by the evaporation area, both devices enabled a continuous and stable evaporation flux through 12 hours of the day simulation with a low value of 0.33 kg $m^{-2}h^{-1}$ for the long stem (due to the higher evaporation area) and an average high value of 0.68 kg $m^{-2}h^{-1}$ for the device with short stem as shown in Fig. 2e main manuscript. It is noteworthy that the bulk water temperature remains almost same for two evaporators and hence there is negligible effect of the stem height on the conducted heat loss from stem to the bulk water. Regarding the salt resistance evaluation, after 12 hours of continuous operation, the salt precipitation was observed only on the edge of the top surface for both evaporators while most of the evaporation area remains clean owing to the fact that capillary pumping was

sufficient to supply liquid water to the edge of mesh leaves (Fig. 2f main manuscript). During the dark cycle, it was noticed that salt crystals fell down from both evaporators, and the mass of peeled salt from the long stem in mangrove-like SVGC was 50% higher with 0.33 g, when compared to the short stem that produced only 0.22 g of salt. However, while increasing the bulk solution salinity from 3.5 wt% to 7 wt%, it was observed that salt crystals tend to accumulate on the solar exposed leaves of SVGC with long stem. The increase of the bulk water concentration reduces the capillary liquid supply (due to increased viscosity) to the leaf and leads the salt to crystalize on the middle of the leaf, as shown in Fig. 2f main manuscript. The comparison in terms of average thermal efficiency is presented in Fig. S11 where the long mesh showed a lower thermal efficiency of 68% when compared to the short mesh with 75%.

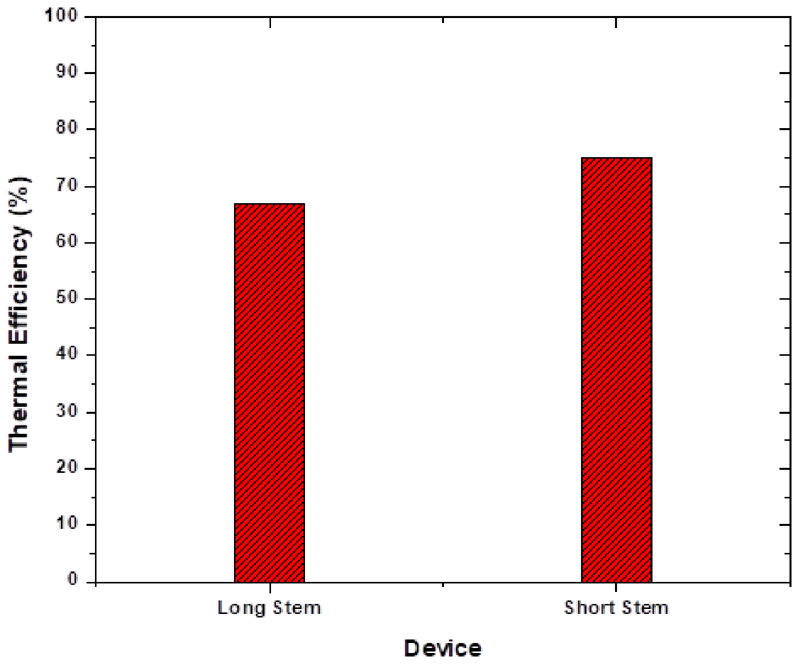

Supplementary Figure 11: comparison between short and long stem in terms of thermal evaporation efficiency

Supplementary Note 5: Freshwater Collection Experiments

Water collection is one of the main applications for solar thermal evaporation. Generally, in all desalination technologies the evaporation process is succeeded by a condensation process in order to collect the evaporated vapor as pure liquid. In our experimental scenario, a transparent spherical chamber was used as a solar still in order to investigate the mangrove-like structure performance as shown in Fig. S12a. To increase the evaporation area, four devices were used in the experiment, and a disposable bowl made from foam that contains real seawater was utilized

as shown in figure Fig. S12a and Fig. S12b. Due to the direct solar thermal heating, the water is evaporated through the titanium meshes and then it condenses once it touches the top glass cover. An insulating foam was kept on the bottom of the chamber to reduce the ground heating as shown in Fig. S12a and Fig. S12b . The proposed device was placed on the rooftop of Khalifa University in Abu Dhabi for outdoor characterization from 10:00 am on June 8th, 2023, to 10:00 am on June 9th, 2023 with a total test period of 24 hours. The measured salinity of the obtained real seawater samples was 4.2 wt% as shown in Fig. S12c. While conducting the experiment, the average direct normal irradiance (DNI) was 878 W m^{-2} during the peak sun hours (10:00 am to 4:00 pm) and the ambient temperature was about 33℃ to 34℃ (Fig. S12d). The results of total dissolved solids (TDS) of seawater before and after desalination are shown in Fig. S12c.

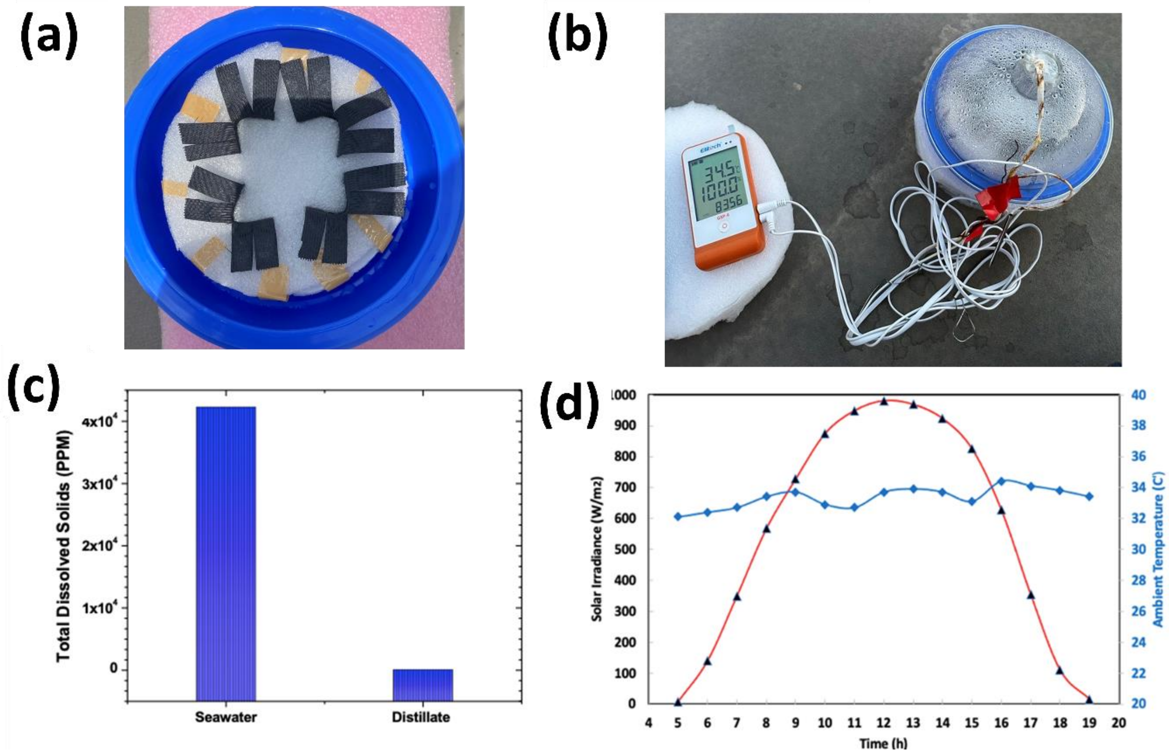

Supplementary Figure 12: Outdoor water collection experiment: (a) Top view of the chamber showing four mangrove mimicked devices used for water collection experiments. (b) Experimental setup on the rooftop of Khalifa university (c) Total dissolved solids (TDS) for the real seawater before and after desalination (d) Outdoor environmental conditions: solar irradiance and ambient temperature at the day of the experiment.

Supplementary Note 6: Stability and Durability Tests

To demonstrate the repeatability of the edge preferred crystallization throughout the day and salt peeling at night, we performed a new series of uninterrupted indoor experiments for a period of four consecutive days. To mimic the natural day/night alternation, the experimental setup was placed for 12 hours under 1-sun simulated solar irradiance and 12 hours under the dark environment as shown in Fig. S13. In all these experiments, we used water with salinity of 3.5 wt%. Our results have revealed that the mass change curve as function of time is stable and linear during the eight cycles of the experiment: four cycles under light and four cycles in dark (Figure S13(a)). Through the first twelve hours of the experiment when the light was on, the water evaporation flux approximately stayed stable in the range of 0.6 kg/m^2 h as depicted in Fig. S13(b). After twelve-hour operation under solar simulator, a layer of a dense salt crust was formed on the edges of the mangroves shaped structure as shown in Fig. S13(c). It is noteworthy that after turning off the simulated sunlight, the evaporation flux significantly decreased to around 0.2 kg m^{-2} h^{-1} (Figure S13(b)). Moreover, it was noticed that the thick salt layer on the edges of the device was self-defoliated and passively peeled off, resulting in ~0.4 g salt per day as shown in Fig. S13(d). Upon switching on the solar simulator again, the evaporation flux was restored, and the salt patches were crystalized on the edges of the tree. The device was able to obtain a similar evaporation flux in the next operation cycle, indicating that our proposed structure is highly stable and reusable without significant deterioration in the evaporation performance or salt production rate as demonstrated by Fig.13(b-c). In our scenario, salt crystallization and accumulation during the day (while the simulated sunlight is on) and automatic passive salt cleaning during the night (under dark environment) will ensure that the device can operate continuously for extended period of time and the solid salt crystals can be peeled regularly without any maintenance or performance degradation.

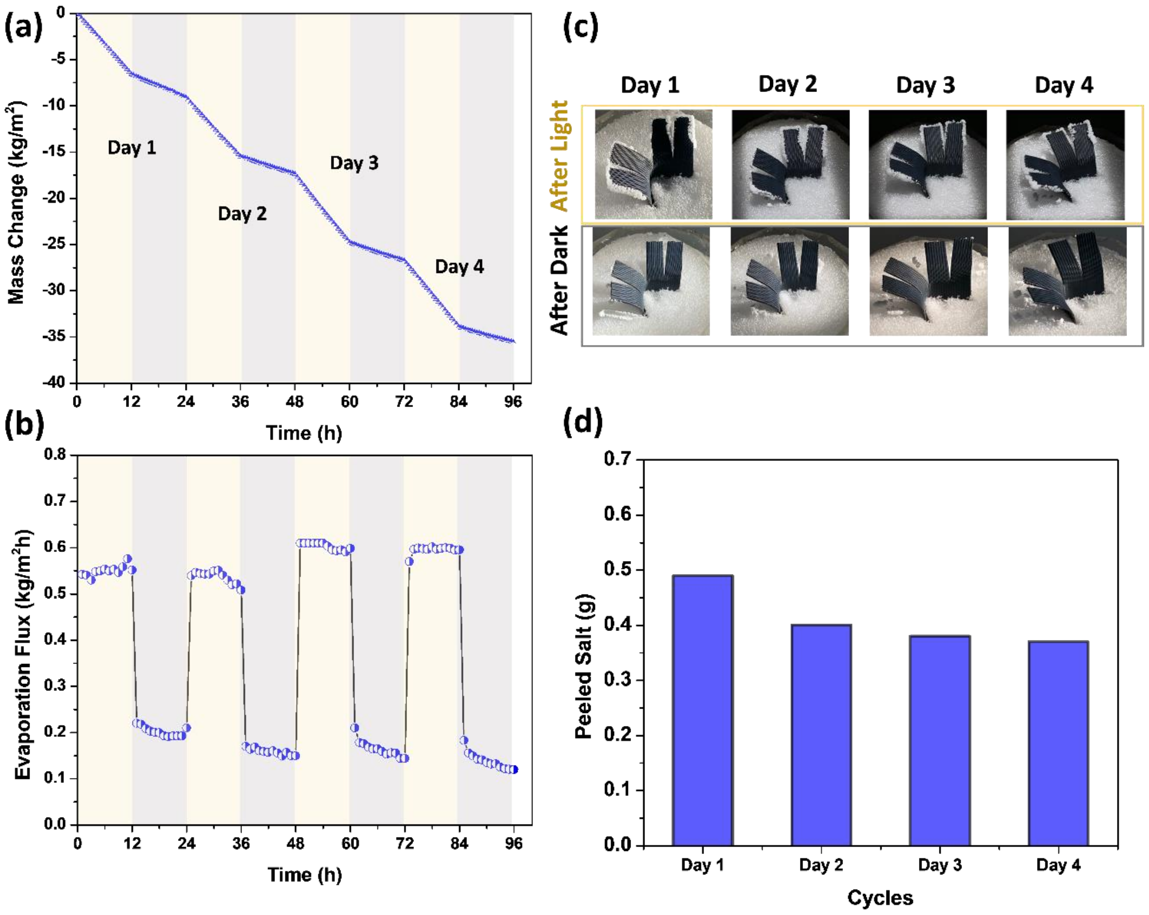

Supplementary Figure 13**:** (a): Indoor experiments: (a-b) measured mass change and evaporation flux for SVGC device consecutive four days. (c) Time laps images for various days showing the device during vapor generation experiments. (d) Mass of collected salt at the end of each day.

The mangrove-mimicked device was also tested under real outdoor conditions on the rooftop of Khalifa University in Abu Dhabi, United Arab Emirates (24.45° N and 53.37° E). The measurements were carried out under clear sky for four consecutive days and nights from the morning of 31st of October 2023 to the night of 3rd of November 2023, as presented in Figure S14 below. While performing the outdoor experiments, the average peak direct solar irradiance for four days was 779.5 W m^{-2} (blue curve in Figure S14a). The ambient temperature varies slightly in the range of 28-30 ℃ over nights and increases during daytime to the peak temperature between 35℃ and 37℃ (red curve in Figure S14a). On the contrary, the ambient relative humidity was found to be higher at night around 70% and low during the day (green curve in Figure S14a).

These experiments were performed with simulated sea water with a salinity of 3.5 wt %. At the end of the first day (6:30 pm), thick salt layers accumulated on the edge of the evaporator, as shown in Fig. S14b. During the nighttime, the salt layers on the edges of the evaporator were passively peeled and fall down on the insulating foam as dry salt. Similar behaviors of SVGC device were found, that is daytime salt accumulation and nighttime passive salt peeling for consecutive four days. The average mass of passively collected salt for four days is 1.18 g/day. In short, edge crystallization during daytime and passive salt peeling at nighttime was occurring alternatively and uninterrupted until all the bulk water got evaporated, as shown by the time lapse images in Figure S14b.

Figure S14c-d shows the water mass change and evaporation rate over of time, recorded on November 1st, 2023. After the sunrise, the mass change was relatively slow as the measured evaporation flux at 8:00 am was 0.55 kg m^{-2} h^{-1}. The mass change curve became steeper after 9:00 am and the hourly measured evaporation flux increased linearly until the peak of 2.2 kg m^{-2} h^{-1} at noon, indicating the high influence of solar irradiance in stimulating the solar vapor generation. Following the sunset, the mass change slope became shallow again and the evaporation flux dropped to about 0.3 kg m^{-2} h^{-1} during nocturnal operation, as presented in Figure S14d. It is worthy to mention that the peak evaporation flux of 2.2 kg m^{-2} h^{-1} during the outdoor experiment was recorded at 780 W m^{-2} (0.78 sun), which is almost four times higher than the indoor evaporation flux of 0.6 kg m^{-2} h^{-1} measured under the irradiance of simulated 1 sun (Fig. S14b). In addition, the average collected mass of salt was 1.18 g/day, almost three times higher than the average collected salt of 0.4 g/day (Fig. S14d) during the indoor controlled experiments. The outdoor experiments indicate the superior impact of heat convection in enhancing both water evaporation and salt production concurrently.

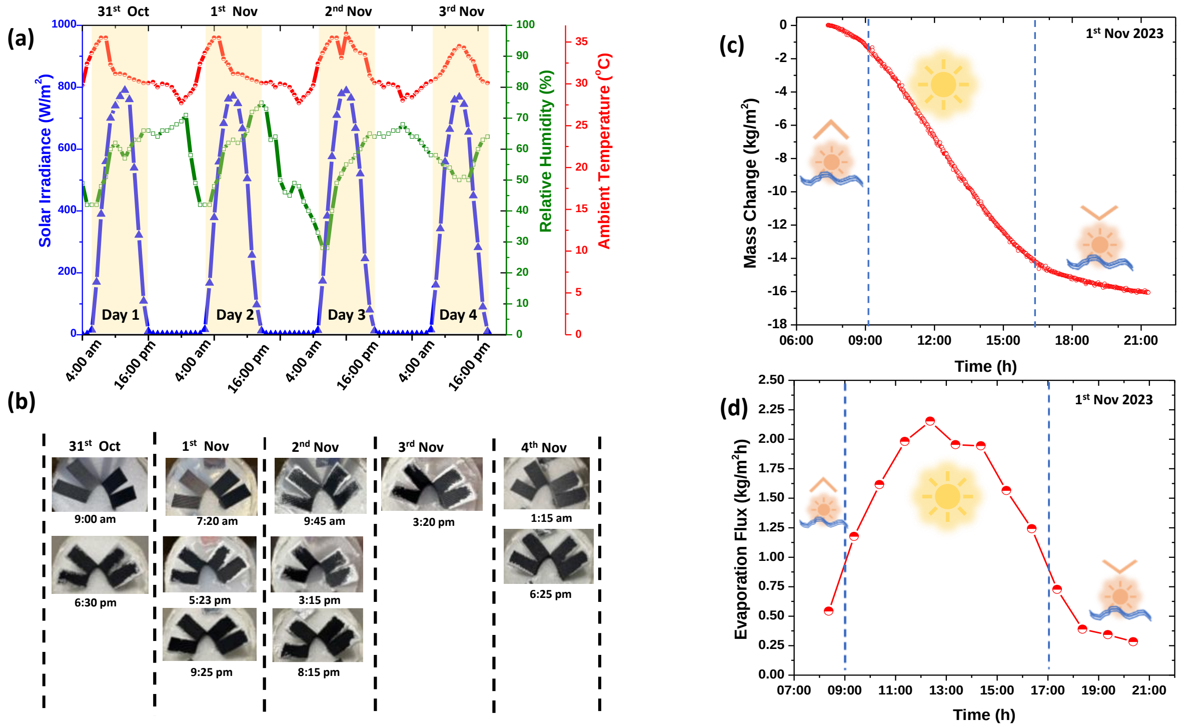

Supplementary Figure 14: Outdoor experiments: (a) Measured solar irradiance, ambient temperature, and relative humidity from Oct 31st, 2023, to Nov 03rd, 2023. (b) Time laps images for various days showing the device during vapor generation and salt collection experiments. (c) and (d) measured mass change and evaporation flux for SVGC device from 7:30 am to 9:00 pm on Nov 1st, 2023.

Supplementary Note 7: Performance Comparison with Literature

We have compared our current work with previous papers in terms of evaporation flux and thermal efficiency. The comparison is listed in Table S1 and Fig. S15 below.

**Materials**	**Evaporation Flux**	**Thermal Efficiency**	**Hours**	**Salt Collection**	**Salinity**	**Ref**
Polyurethane, Polystyrene foam & black paint	-	86	6	No	20	[4]
Self-assembled aluminum nanoparticles	0.93	57	1	No	2.75	[5]
Femtosecond laser rendered metal panel	1.26	67	1	No	3.5	[6]
Fabric wick polystyrene	-	55	0.55	No	3.5	[7]
Filter paper-CNTs	1.05	81	600	Yes	3.5	[8]
CuS-coated PE membrane	1.02	63.9	1	No	0	[9]
Copper-silicon nanowire porous membrane	0.81	50.9	1	No	3.5	[10]
Ppy-coated Hydrophilic PVDF membrane	0.92	54.3	2.33	no	3.5	[11]
Electrospun CB/PMMA-PAN Janus absorbers	0.92	51	-	no		[12]
Janus vertically oriented porous membranes	1.08	62.8	-	no	20	[13]
Janus SiO_2/cellulose nanofiber/carbon nanotube	1.2	80%	100	no	3.5	[14]
Poly-pyrrol and polyvinyl alcohol (ppy/PVA)	1.09	75	60	Yes	3.5	[15]
This Work **	1.22	94	12	Yes	3.5	

Supplementary Table 1: Comparison for various materials used as SVG in terms of evaporation flux & thermal efficiency.

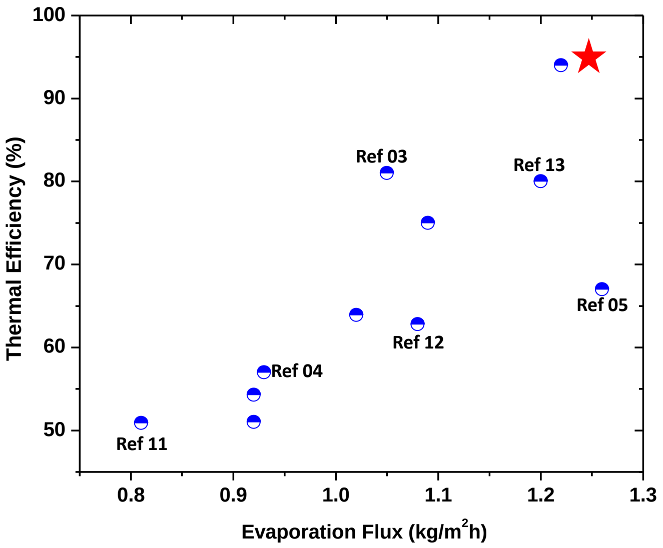

Supplementary Figure 15: Comparing the thermal efficiency and evaporation flux of this work to the reported literature.

Supplementary Note 8: Comparing the specific requirements from internationally recognized authority.

Following Table 2 demonstrates the capability of our proposed device and potential in passive freshwater production according to Oman Humanitarian Desalination Challenge (OHDC).

Requirements	Detailed Requirements	Proposed device potential
Hand-held	The device should be hand-held and easily transportable	The SVGC device is compact, light, foldable, thus suitable for mobile applications
Low-cost	The estimated production cost of the device should be 20 $	The cost of our SVGC device is about 2 $ per unit, therefore water productivity can be greatly enhanced if 10 units are used.
Robust	Resilient, corrosion resistant operated through pictorial instructions, long shelf-life, minimal use of easily lost parts	The SVGC device made of titanium dioxide meshes (TiO_2) is intrinsically anti-corrosive, strong and flexible
Rate of Production	Device should produce a minimum of 3 liters of purified water per day including cloudy days	Our findings show that the device is able to produce 2.2 L/m^2day when using 4 units in real outdoor conditions. Thus, it is expected to collect over 5 L/day in sunny day when using 10 units, beyond 3 L/day even in cloudy days, at a fixed cost of 20 $
Stand-alone	There should be no addition of chemicals, fuels, or other external materials, other than the seawater to be purified.	We are using the real seawater directly for freshwater production without any pre-treatment or chemical additives.
Short-term use	The device should operate for a minimum of 30 days.	The proposed device was tested for more than two years since we started this work without considerable efficiency drop
Quality	Device should purify 100 NTU, 35,000 mg/L seawater to 1000 mg/L TDS and meet WHO maximum contaminant levels.	The device is able to purify gulf real seawater with a salinity of 42,000 mg/L to water with a low salinity of 200 mg/L, which meets the WHO requirements well.

Supplementary Table 2: Comparison for various materials used as SVG in terms of evaporation flux & thermal efficiency.

Supplementary Note 9: Crusty and Patchy salt growth

In this work, we found that the patchy salt as represented in Fig. S16 a and b can promote and enhance the evaporation of water vapor while the crusty salt as shown in Fig, S16 c and d can hinder the evaporation process by blocking the pores.

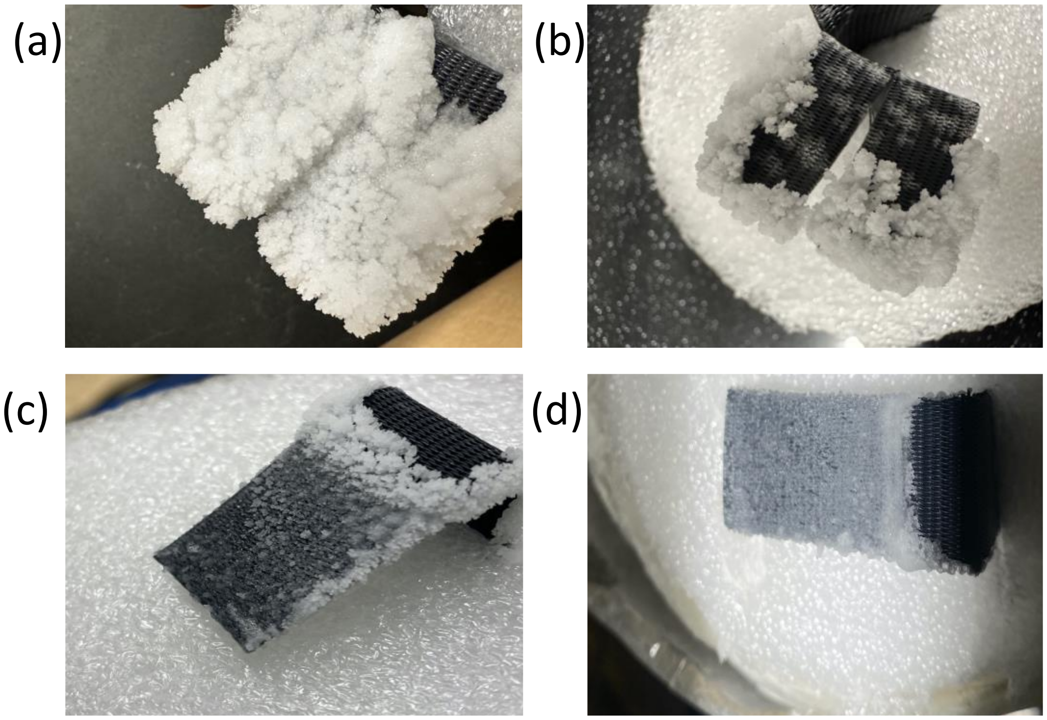

Supplementary Figure 16: Different Morphology of the salt crystals: (a) and (b) Patchy salt crystals while (c) and (d) are showing Crusty salt crystals.

Supplementary Note 10: Theoretical analysis to predict stem length for edge-preferred crystallization.

In this section, we have provided the theoretical analysis of edge-preferred crystallization in the mesh based on thermodynamics:

Thermodynamic Analysis of the Edge Crystallization

When using TiO_2/Ti mesh-based solar vapor generator and crystallizer (SVGC), the salt crystallization behavior observed in experiments results from water evaporation, as shown in Figs. S17-18. In general, the classical nucleation theory can be used to investigate the change in free energy for homogenous nucleation of salt crystals that is expressed by:[16]

$$\Delta G_{\text{hom}} = -\frac{4}{3}\pi r^3 \rho_s \Delta\mu + \pi r^2 \gamma_{lc} \qquad \text{(S7)}$$

The first term on the right side of Eq. (S7) represents the free energy change due to phase transition, while the second term shows the energy change due to creation of interface between the salt solution and newly forming crystals. r is the radius of the spherical nucleus of new phase, ρ_s is number density of crystals, $\Delta\mu$ is the difference in chemical potential of solute (i.e., NaCl salt) in solution between supersaturated and saturated states. γ_{lc} is the interfacial tension between liquid phase (i.e., solution) and crystal. In fact, the salt nucleation on titanium mesh surface is heterogeneous. For heterogeneous nucleation on a flat substrate, Eq. (S7) is modified as:[16]

$$\Delta G_{het-flat} = -\frac{2}{3}\pi r^3 \rho_s \Delta\mu + \pi r^2 \left(2\gamma_{lc} + \gamma_{sc} - \gamma_{ls}\right) \qquad \text{(S8)}$$

The variables γ_{sc} and γ_{ls} refer to the interfacial tensions between substrate-crystal and liquid-substrate, respectively.

The individual wire of titanium mesh used for our SVGC device is covered with nano/micro-structured titanium dioxide after chemical etching (Fig. 1c in the main manuscript). In addition, the mesh exhibits nearly cone-shaped cavities due to the weaving pattern of wires (Fig. S18 d-e). Both the nano/microstructure and cavities will impact the energy barrier for nucleation of salt crystals. The cone angle of the cavities between shute wires illustrated in Fig. S18e is around ~ 60°. The liquid bridge between two extended wires (Fig. S18 c-d) can also be approximated as cone-shaped cavity though spacing between wires could impact the shape of the liquid bridge. For cone-shaped cavities and heterogeneous nucleation, Eq. S8 can be modified as:[17]

$$\Delta G_{het-cone} = -\frac{4}{3}\pi r^3 \rho_s \Delta\mu + \pi r^2 \gamma_{lc} \left[\frac{1}{4}\left(2 - 3\sin\left(\theta + \frac{\psi}{2}\right) + \sin^3\left(\theta + \frac{\psi}{2}\right) - \cos^3\left(\theta + \frac{\psi}{2}\right)\cot\left(\frac{\psi}{2}\right)\right)\right] \qquad \text{(S9)}$$

ψ is the cavity angle while θ is the interfacial angle between solution and nucleated phase as illustrated in Fig. S18e. There are three important parameters in Eq. (S9): $\Delta\mu$, θ and ψ. The impact of these parameters on crystallization behavior of our biomimetic SVGC is analyzed systematically in the subsequent sections.

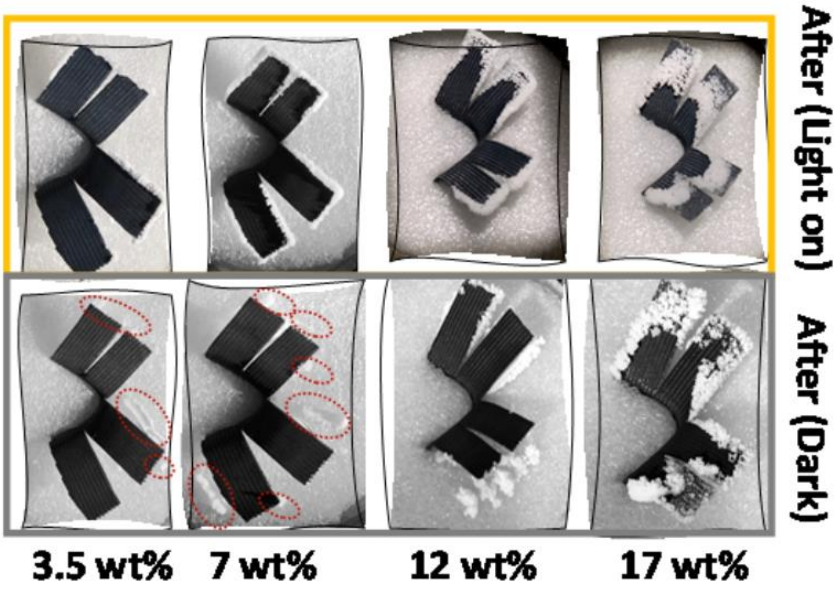

Supplementary Figure 17: Optical images for bio-mimicked SVGC after one day cycle (yellow box) and after one night cycle (grey box) using water of various salinities.

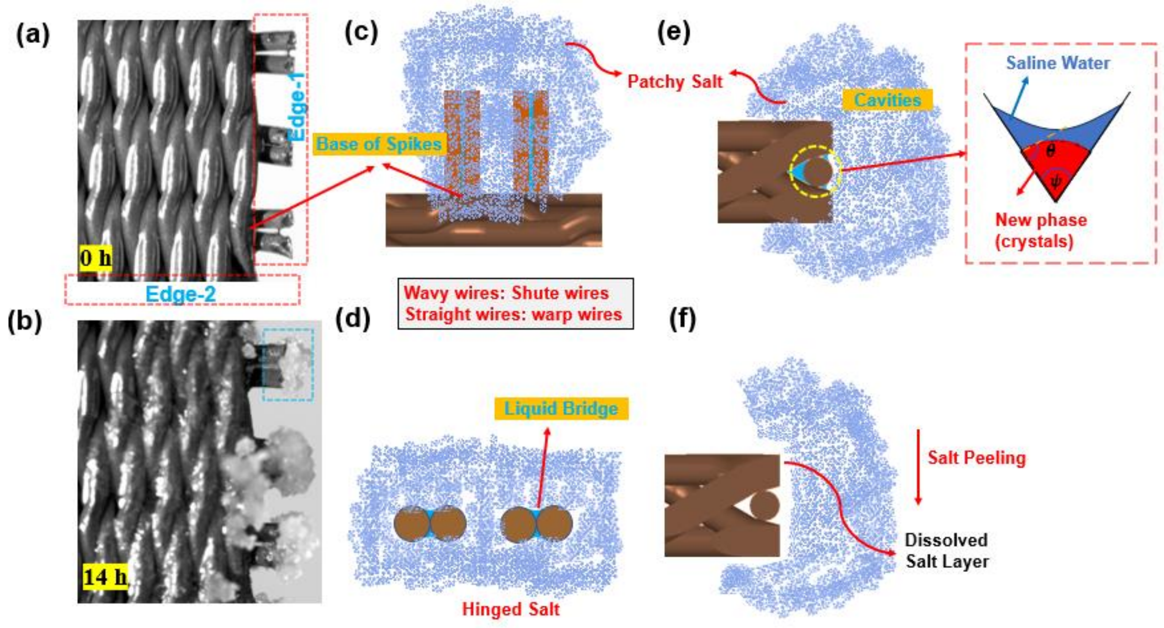

Supplementary Figure 18: Salt precipitation on extended warp wires of SVGC at (a) t =0 h and (b) t =14 h under ambient conditions. Illustration for the pattern of salt precipitation on the extended warp wires from (c) top and (d) side views. (e) Growth of salt perpendicular to the

direction of shute wire and (f) illustration for the salt peeling. The dimensions of the extended warp wires and shute wires are exaggerated for clarity in the schematic given in Fig. (c-f). Figure e also illustrates the nucleation of new phase inside the cone-shaped cavity containing saline water.

Effect of Chemical Potential Difference ($\Delta\mu$)

Under low super saturation at constant temperature and pressure, the chemical potential difference for evaporative crystallization of individual species is given by:[18]

$$\Delta\mu = RT\sigma = RT\left(\frac{c}{c_{eq}} - 1\right) \tag{S10}$$

where R is the universal gas constant, T is the absolute temperature and σ is the relative supersaturation. Similarly, c and c_{eq} are actual and equilibrium (or saturated) salt concentration in solution, respectively. An increase in NaCl concentration will enhance the chemical potential difference of salt in the solution (Eq. S10), thus reducing the energy barrier for salt nucleation (Eq. S9). In our work, the concentration of salt along the mesh length is affected by the evaporation-driven transport of saline water. Therefore, it is important to predict the locations where the concentration of salt exceeds the saturation value and the salt crystallization (i.e., nucleation) occurs most likely. Based on numerical simulation, a detailed analysis of the concentration profile during evaporation along the evaporator length is provided in the following subsection.

COMSOL Simulation of Variation in Salt Concentration:

In order to study the variation in salt concentration along the stem of SVGC, we carried out the COMSOL simulation for the spatial salt distribution on porous evaporator. The computational domain for the numerical analysis is shown in Fig. S19. A coupled phenomenon of liquid and species transport was modelled by employing the “Darcy Law” and “Transport of Diluted Species” Modules of the COMSOL. The corresponding mass conservation for the liquid transport is written as:

$$\frac{\partial}{\partial t}(\phi\rho) + \nabla.(\rho u) = Q_m \tag{S11}$$

The first term on the left is related to temporal change in mass while the second term represents advective transport of mass driven by evaporation. Q_m is the source term for evaporation (J_v) which is related to the evaporation flux (J_v) and thickness of the mesh (b) as: $Q_m = -J_v / b$. ϕ is the porosity of meshes while ρ is the density of salt water. The liquid transport through the mesh is driven by evaporation and enabled by capillary wicking, which can be modelled through Darcy Law:

$$u = -\frac{K}{\hat{\mu}}(\nabla p - \rho g) \tag{S12}$$

where K is the permeability of meshes (~ 60 μm^2), $\hat{\mu}$ is the viscosity of salt water and p is the pressure. The conservation equation for species transport in saturated porous media can be written as:

$$\phi \frac{\partial c}{\partial t} + \nabla.\left(-\phi \frac{D_e}{\tau} \nabla c\right) + u.\nabla c = S \tag{S13}$$

The first term on the left side of Eq. (S13) represents transient variation in concentration profile while the second term is related to the back diffusion of salt. Similarly, the third term represents the advective transport of salt. τ is the tortuosity of the evaporator while D_e is effective diffusion coefficient of the evaporator. S is the source term for salt concentration and related to evaporation as: $S = -Q_m c / \rho$. Note that Q_m is a negative quantity while S is positive.

Initial and Boundary Conditions:

A constant salt concentration as the inflow boundary condition is applied at Boundary 1 (Fig. S19). At Boundaries 2, 3 and 4, an evaporation flux is applied when considering the contribution from SVGC edges (with free evaporation to the surrounding), and no evaporation flux is considered in the other case without edge effect. As an initial condition, the domain was assumed to be saturated with the concentration of inflow (i.e., bulk solution). The concentration-dependent properties (i.e., density and viscosity) of the saline water are employed in the simulation. A mesh convergence analysis was performed to ensure that results are independent of the grid size.

Assumptions:

1. The back diffusion is neglected owing to the fact that Peclet number is significantly higher than 1 (which means advective transport dominates the diffusive one). Here, the aim is to obtain concentration profile for evaporator with zero liquid discharge rather than salt-resistant evaporator with back diffusion. Most importantly, the steady-state solution for the concentration profile cannot be obtained if the back diffusion is considered.

Thus, only two important competing factors remain to shape the concentration profile: the evaporation-driven advection (transport) of salt ion is countered by the salt generated from precipitation (represented by the source term S), both owing to evaporation. The boundary/location at which both factors are balanced will correspond to c/c_{sat}=1.

2. The effect of gravity is neglected.

It is pertinent to mention that the precipitation process is not a steady-state process but rather a transient one. Though we are making these critical assumptions to obtain a steady-steady solution, these assumptions do not affect the accuracy and validity of the simulation results.

3. Evaporation flux is considered uniform over the evaporator surface unless stated.
4. Porous medium (mesh) is homogeneous, and its properties are uniform through the porous matrix.
5. The temperature of the evaporator is uniform.

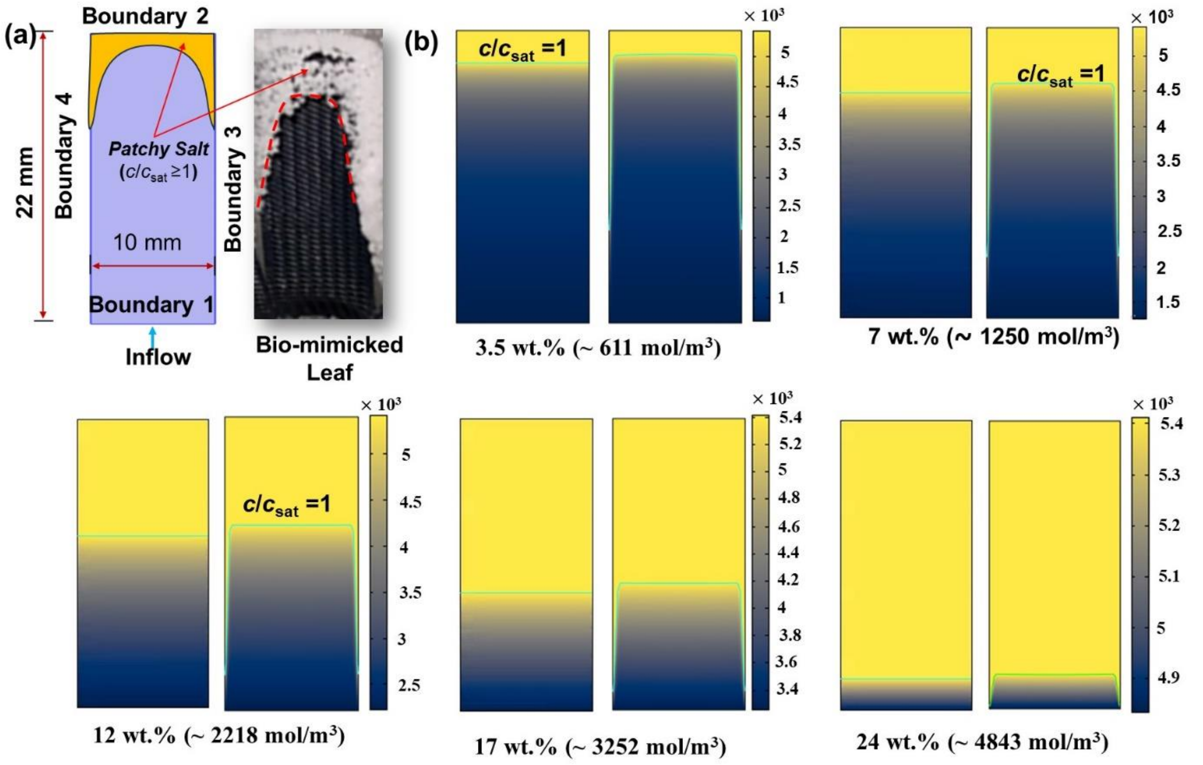

Supplementary Figure 19: (a) Computational domain for the COMSOL simulations. (b) Contour map for effect of salinity on salt concentration for two cases i.e., with and without edge effect by using water of various salinities. The black line/curve represents c/c_{sat} =1.

The simulation results for spatial variation of salt concentration along the evaporator length are shown in Fig. S19. It can be seen that the salt concentration increases along evaporator length and reaches the saturation value (i.e., 26.3 wt.% or ~5411 mol/m^3) at a certain distance from the inlet (i.e., Boundary 1). As the bulk (or inflow) concentration increases, the length of the probable crystal-free region is reduced. This is applicable to both cases i.e., with and without the edge effect. Figure S20 shows variation in dimensionless salt concentration (c/c_{sat}) as a function of precipitation-free length to the total evaporation length (l_{sat} / L). In the case of no edge effect, the predicted l_{sat} /L values corresponding to c/c_{sat} = 1 for 3.5, 6, 12, 18, and 24 wt.% are 0.89, 0.77, 0.6, 0.40, and 0.11, respectively. The l_{sat} / L values become larger when the edge effect is introduced, leading to more crystal-free regions. These

simulation results in Fig. S20b are consistent with the experimental results for salt precipitation with our bio-mimicked SVGC. The deviation between experimental l_{sat} / L (determined based on centerline of the mesh) and simulated l_{sat} /L is due to several reasons. First, the role of back diffusion is neglected in COMSOL simulations. Secondly, the edge of the mesh would provide additional surface for salt precipitation. It should also be noted that crystallization may not necessarily occur at c/c_{sat} =1 rather at higher values. For instance, nucleation took place at the supersaturation of around c/c_{sat}=1.6 for the case of glass capillaries filled with NaCl saline solution, as reported by Shahidzadeh et. al.[19] Nevertheless, the simulation results provide good estimate for the locations where c/c_{sat} is higher than 1 since these locations are more prone to crystallization, owing to the fact that the energy barrier for the nucleation of salt will be reduced at locations where c/c_{sat} > 1 (Eq. S9).

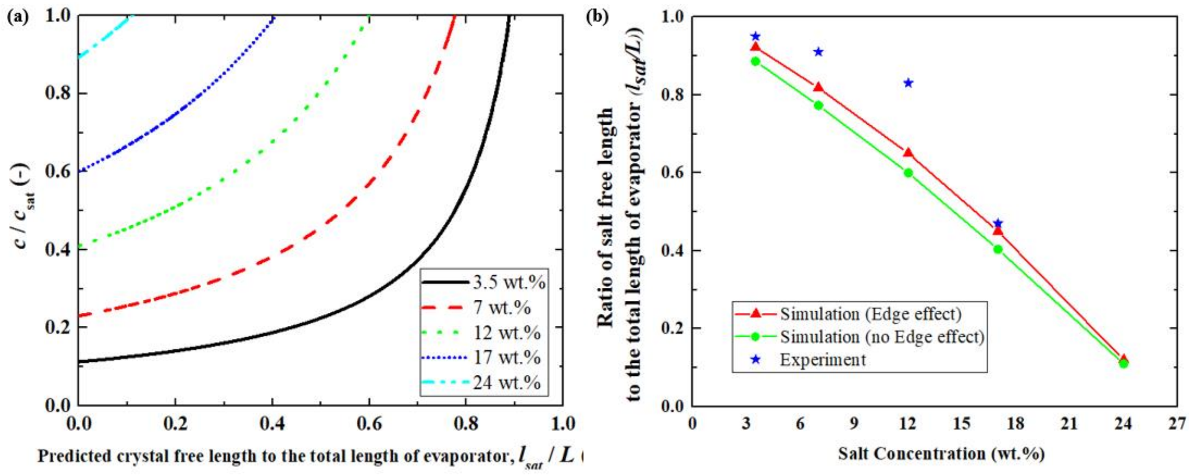

Supplementary Figure 20: COMSOL simulation results for variation in crystal free length to the total evaporation length (l_{sat} / L) as a function of dimensionless salt concentration (c/c_{sat}). (b) Comparison of simulation results with experimental data for the ratio of salt free length to the total length of evaporator (l_{sat}/L) corresponding to c/c_{sat}=1.

Effect of Micro-Structure and Weaving Pattern

Based on Eqs. (S7) and (S9), the ratio of heterogeneous to homogeneous nucleation energy barrier (ΔG^{*}) can be written as:

$$\Delta G^{*}=\frac{\Delta G_{het-cone}}{\Delta G_{\hom}}=\left[\frac{1}{4}\left(2-3\sin\left(\theta+\frac{\psi}{2}\right)+\sin^{3}\left(\theta+\frac{\psi}{2}\right)-\cos^{3}\left(\theta+\frac{\psi}{2}\right)\mathrm{co}t\left(\frac{\psi}{2}\right)\right)\right] \quad \text{(S14)}$$

For heterogeneous nucleation in a conic cavity, the new phase (salt) will form an angle θ with the substrate (mesh wire). Owing to the intricate and complex nature of the nucleation phenomena, θ can vary from 0º to 180º. For heterogeneous nucleation on flat substrate, the cavity angle of ~ 180º is also considered in the analysis. Fig. S21 shows that ΔG^{*}< 1, which means the energy barrier for

heterogeneous nucleation is lower in comparison with homogeneous one in the whole range of θ. Also, nucleation is more probable to occur in the cavity compared to the flat substrate surface since ΔG^* for β =180º is higher than that for β =60º. Based on the results given in Fig. S21, it can be postulated that the nano/micro structures (as observed in SEM images shown in Fig. 1d in the main manuscript) on the mesh wire will have lower energy barrier compared to the homogeneous case. The overall effect of the low energy barrier, which facilitates nucleation/crystallization, will be the same at all locations of the evaporator, while crystallization is not uniform over the evaporator surface. This clearly demonstrates the dominant impact of chemical potential difference of salt in solution on crystallization, compared with the reduction in nucleation energy barrier due to micro-structure and cavities (quantified through θ and ψ). Moreover, the precipitation behavior observed in experiments is in consistency with the concentration profiles obtained through COMSOL simulation, which confirms the important role of supersaturation. In fact, some questions remain, for instance, why do we observe quick crystallization at the extended spikes (i.e., wires at Edge-1 in Fig. S18a) compared to non-spike edge of the evaporator (Edge-2 in Fig. S18a)? Is it because of low nucleation energy barrier due to the reduced ΔG^*, supersaturation or something else? As stated before, supersaturation plays a more important role than ΔG^*. Just like the main evaporator surface (where we have l_{sat}/L defined by c/c_{sat} =1 as given in Fig. S19-20), concentration will also increase along the spike's length and saline water at the tip of spikes will reach the supersaturation state earlier than that at the spike base (Fig. S18a-b). Therefore, higher supersaturation at the spike tip will increase chemical potential difference ($\Delta\mu$) of salt, thus reducing nucleation barrier. However, crystallization behavior will not be same on all spikes, because it also depends on the distance of a particular spike from the evaporator inlet, which affects salt concentration at the base of the spike. Spikes far from the evaporator inlet (boundary 1, Fig. S19a) will have high concentration at their base (as marked in Figs. S18 a,c), so these spikes will have more salt crystallization in comparison with the ones near the evaporator inlet (as observed in Fig. 5a-b in the main manuscript). In addition, the evaporation rate around the spikes is higher than that on the main evaporator surface (dark region in Fig. S19a) since it is easier for vapor diffusion into air near spikes. Moreover, the liquid film at/between the spikes could be thinner than that at the main surface, and higher evaporation at thin film region will lead saline water to reach supersaturation (c/c_{sat}) more easily, hence more crystallization.

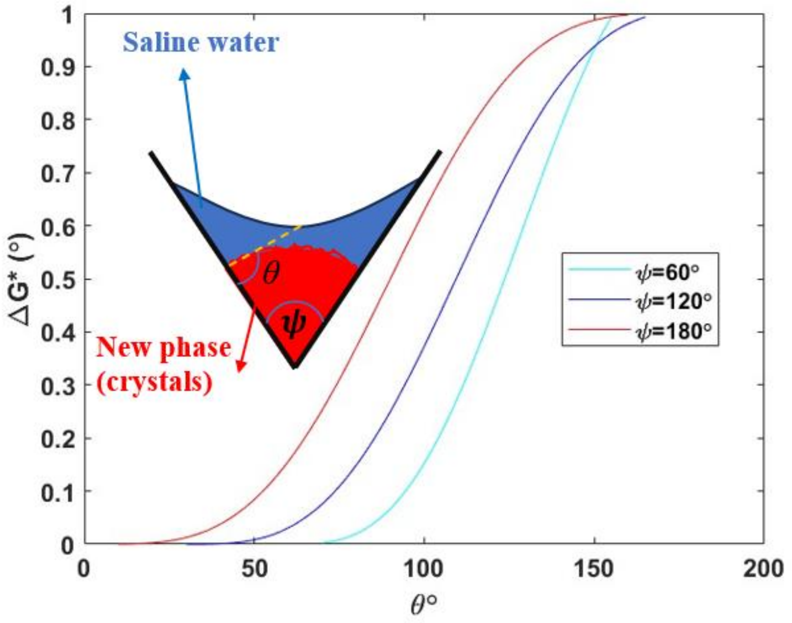

Supplementary Figure 21: Effect of interfacial angle θ and cone-shape cavity angle (ψ) on dimensionless energy barrier for nucleation.

Supplementary Note 11: Estimation of Back Diffusion Coefficient

We have conducted both experimental study and numerical (finite-element) simulation to estimate the back diffusion coefficient of salt (i.e., NaCl) as shown in Fig. S22. The experimental setup along with simulation domain for the estimation of back diffusion coefficient contains single leaf of our SVGC device as shown in Fig. S22. The leaf was dipped in saline solution of 3.5 wt.% (Fig. S22a), and the vertical section of the leaf is of 2.2 cm in length while titled one is of 2.4 cm (Fig. S22b). The whole setup was kept in a close chamber to keep the humidity around ~ 100% to avoid evaporation. A very small droplet of saline water was taken from the evaporator at z =22 mm to measure the salt concentration with refractometer. The experiments were repeated three times to improve the accuracy with a sampling interval of 1 hour.

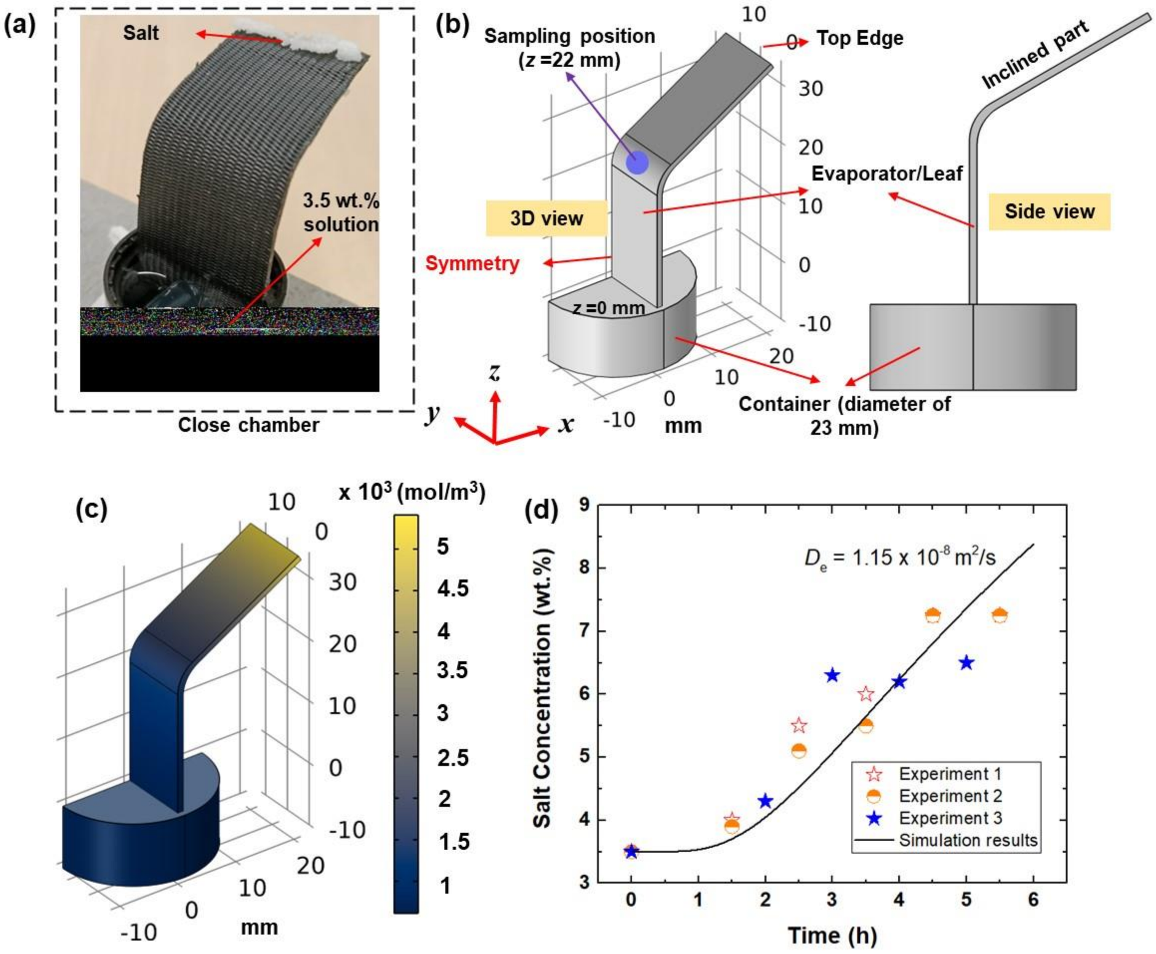

Supplementary Figure 22: Experimental setup for the estimation of back diffusion coefficient with corresponding (b) computational domain for COMSOL simulations. (c) Volumetric concentration profile at $t = 6$ h in mol/m^3, (d) Variation in salt concentration (wt.%) over time at z = 22 mm. The solid black line is the simulation data while scatter data points are of experiments.

Transient simulation was performed with COMSOL Multiphysics to investigate the diffusion process and estimate the corresponding diffusion coefficient. A 3D model coupling "Darcy Law" and the "Transport of diluted species" is developed to study the back diffusion process. The dimensions of the computational domain are in accordance with the experiments (Fig. S22b). Saline water (3.5 wt.%) in the container, which is in contact with the base of porous evaporator, was modelled as porous media with porosity of 1 and effective permeability equivalent to a pipe (i.e., $R^2/8$, where R is container radius). Standard value of diffusion coefficient (~ 1.5 x 10^{-9} m^2/s) was considered for the saline water in container, while the diffusion coefficient for the leaf was adjusted so that the simulated concentration profile matches the experimental data. Bruggeman model was employed to calculate effective

diffusion coefficient as a function of tortuosity and porosity. Thermophysical properties of the saline water and governing equations are the same as those in previous simulation (for the response of comment#1). Dirichlet boundary condition is applied at the top edge of the evaporator (Fig. S22b) with fixed concentration of 26.3 wt.%, reproducing the concentration of saline water in-contact with the patchy salt. The leaf is assumed to be initially saturated with 3.5 wt.%. No mass flux boundary conditions ($-n.\rho u$=0 and $-n.(D\,\nabla c)$=0) are applied at all other surfaces except symmetric boundary condition (Fig. S22b) and top edge of the leaf.

Figure S22c shows volumetric contours profile for salt concentration after 6 hr of back diffusion while Fig. S22d reports experimental and simulation results for the variation in salt concentration with time at z = 24 mm. Diffusion coefficient based on curve fitting of the experimental data with the simulation results for purely molecular diffusion is found to be ~ 1.15 x 10^{-8} m^2/s. The magnitude of the diffusion coefficient is one orders of magnitude higher than the standard diffusion coefficient (~ 1.5 x 10^{-9} m^2/s). It is important to mention that the mechanism of back diffusion could be different from the simple purely molecular diffusion as natural convection and Marangoni effect will induce velocity gradients. The value of back diffusion coefficient (~1.15 x 10^{-8} m^2/s) could have been under-estimated since the circulation of saline water (owing to the natural convection and Marangoni effect) may result in non-uniform concentration profile along the horizontal direction of the evaporator (along x-y plane as shown in Fig. S22b), which could also influence the concentration of saline water at base of the leaf (z =0 mm as shown in Fig. S22b). Surprisingly, we observed small difference in bulk concentration at z =0 compared to the one at z =22 mm. This implies the presence of natural convection and Marangoni effect in addition to the purely molecular diffusion. Thus, the actual diffusion coefficient could be higher than the above estimates (i.e., ~1.15 x 10^{-8} m^2/s) as reported in. [20]

The surface tension of the saturated saline water (26.3 wt.%, namely 5411 mol/m^3) is 84 mN/m, which is 1.15 times of the surface tension of pure water, involving total variation of ~ 18% [21]. Similarly, more than 20% variation in density exists between pure water (with density of 997 kg/m^3) and saturated saline water (with density of ~ 1200 kg/m^3). [22] Therefore, both the density-driven (gravity) and surface tension-driven (i.e., Marangoni effect) flow influence the effective back diffusion coefficient of salt for our SVGC device. The saline water at the top edge of the evaporator (shown in Fig. S22a) will have higher surface tension as well as higher density compared to the saline solution at the base of the evaporator (i.e., bulk solution). A surface

tension gradient along the length of the evaporator (owing to concentration gradient) may induce liquid circulation and assist the back diffusion process as observed by Morciano et al.[23]

It is worth mentioning that our SVGC device has two different types of edges, depending upon the weaving directions. The warp wires are parallel to the length of the evaporator (along *z* direction) while they are perpendicular to the top edge of the evaporator (i.e., perpendicular to x-y plane). These weaving patterns with different edge structure will affect the porosity and tortuosity of the leaf. There will be a small micro-channel of 270 µm (which is equivalent to wire diameter) when warp wire illustrated in R2e is removed from the leaf. The porosity of the resulting micro-channel will be almost zero. As effective diffusion coefficient depends on porosity, it will be higher for this specified micro-channel compared to the middle surface of the leaf. These potentially differing local values of diffusion coefficient will introduce density and surface tension gradient, thus leading to density driven and Marangoni driven flow.

Supplementary Note 12: Characterization of wicking through patchy salt.

With the help of the IR imaging, we also characterized the liquid propagation through porous and patchy salt, where the patchy salt with a length of 5 mm precipitated at the edge of the stem (Fig.4 main manuscript) was used for this purpose. Time-lapse IR images with an interval of 5 seconds in the salt rewetting process are shown in (Fig.4 main manuscript). The evolution of the liquid front is distinguishable in the IR ranges, and the wetted salt is represented by blue while the dry salt is represented by orange color. In addition, the direct IR readings as a function of position in the salt for the different time lapses are also reported in Fig.S23a. The sudden jumps in an IR reading versus distance curve indicate that this position is the wicking front. Based on these IR images, we managed to determine the distance of the water propagation inside the salt as presented in the top subplot of Fig.S23b. It can be observed that the velocity of water inside the salt is not linear with an average value of ~ 0.23 mm/s (bottom subplot of Fig.S23b). These results confirm the patchy nature of the salt is indeed a contributing factor for enlarging evaporating interface and surface area for vaporization.

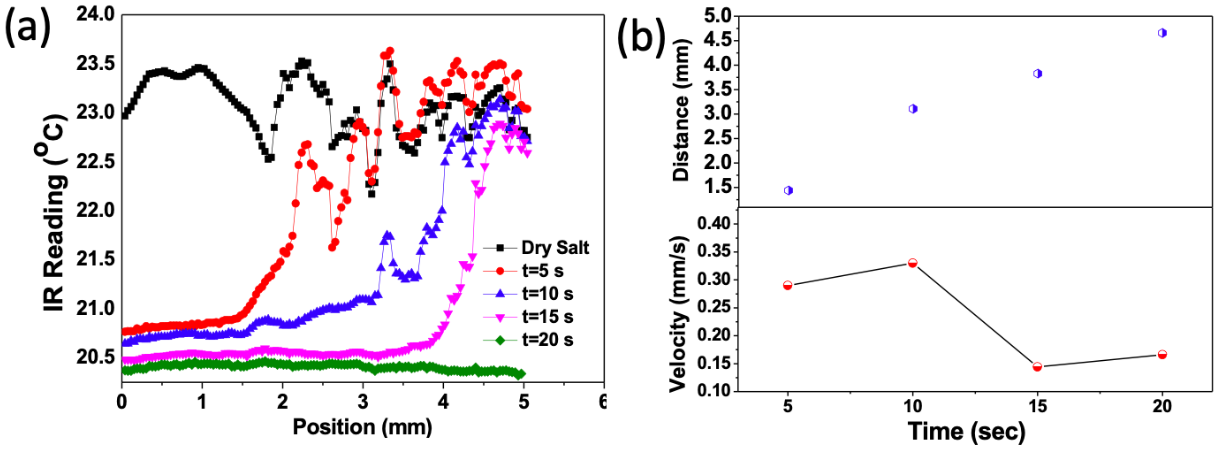

Supplementary Figure 23: Liquid propagation through the salt recorded by IR camera: (a) IR readings along the salt for different time intervals. (b) The corresponding wicking distance and wicking velocity in salt overtime.

Supplementary Note 13: Infrared images for the SVGC under Different solar light irradiation

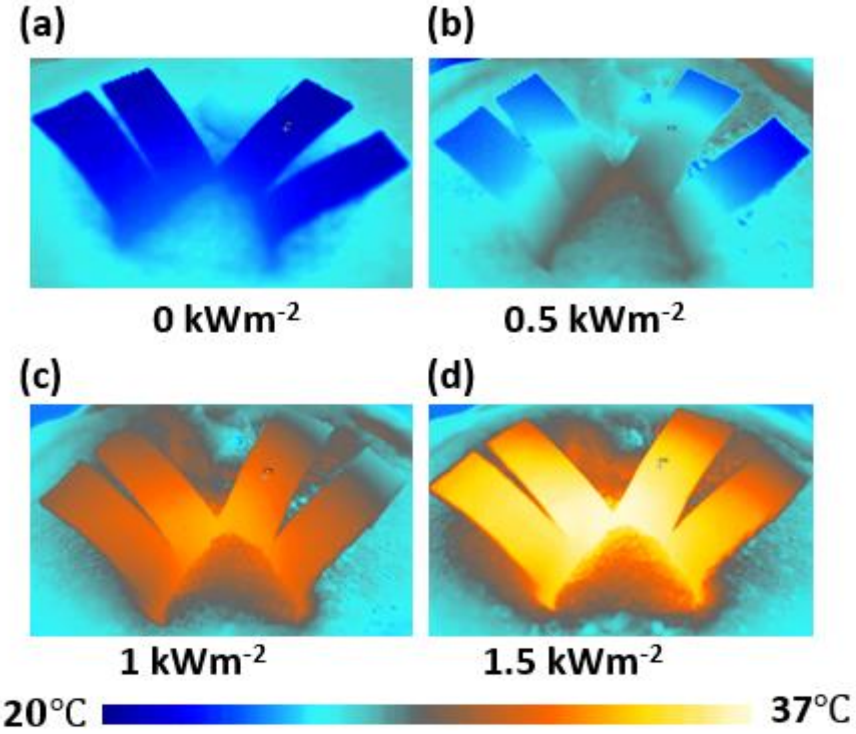

Supplementary Figure 24: respective IR images of tree-like SVGC under various intensities of light illumination (a) 0 kWm^{-2} (0 sun) (b) 0.5 kWm^{-2} (0.5 sun) (c) 1.0 kWm^{-2} (1.0 sun) (d) 1.5 kWm^{-2} (1.5 sun). Reproduced with permission from [1].

Supplementary References:

1. Abdelsalam, M. A., Sajjad, M., Li, H., AlMarzooqi, F. & Zhang, T. DIRECT SOLAR THERMAL DISTILLATION WITH FLOWER-MIMICKED VAPOR GENERATOR FOR FRESHWATER COLLECTION. in *Proceeding of International Heat Transfer Conference 17* 10 (Begellhouse, 2023). doi:10.1615/IHTC17.400-90.

2. Agrawal, Y. *et al.* High-Performance Stable Field Emission with Ultralow Turn on Voltage from rGO Conformal Coated TiO2 Nanotubes 3D Arrays. *Sci Rep* **5**, 11612 (2015).

3. Liu, H. *et al.* Sunlight-Sensitive Anti-Fouling Nanostructured TiO2 coated Cu Meshes for Ultrafast Oily Water Treatment. *Sci Rep* **6**, 25414 (2016).

4. Zhang, L. *et al.* Highly efficient and salt rejecting solar evaporation via a wick-free confined water layer. *Nat Commun* **13**, 849 (2022).

5. Zhou, L. *et al.* 3D self-assembly of aluminium nanoparticles for plasmon-enhanced solar desalination. *Nat Photonics* **10**, 393–398 (2016).

6. Singh, S. C. *et al.* Solar-trackable super-wicking black metal panel for photothermal water sanitation. *Nat Sustain* **3**, 938–946 (2020).

7. Ni, G. *et al.* A salt-rejecting floating solar still for low-cost desalination. *Energy Environ Sci* **11**, 1510–1519 (2018).

8. Xia, Y. *et al.* Spatially isolating salt crystallisation from water evaporation for continuous solar steam generation and salt harvesting. *Energy Environ Sci* **12**, 1840–1847 (2019).

9. Shang, M. *et al.* Full-Spectrum Solar-to-Heat Conversion Membrane with Interfacial Plasmonic Heating Ability for High-Efficiency Desalination of Seawater. *ACS Appl Energy Mater* **1**, 56–61 (2018).

10. Song, X. *et al.* Omnidirectional and effective salt-rejecting absorber with rationally designed nanoarchitecture for efficient and durable solar vapour generation. *J Mater Chem A Mater* **6**, 22976–22986 (2018).

11. Wang, Y. *et al.* Improved light-harvesting and thermal management for efficient solar-driven water evaporation using 3D photothermal cones. *J Mater Chem A Mater* **6**, 9874–9881 (2018).

12. Xu, W. *et al.* Flexible and Salt Resistant Janus Absorbers by Electrospinning for Stable and Efficient Solar Desalination. *Adv Energy Mater* **8**, (2018).

13. Yu, H.-H. *et al.* Janus Poly(Vinylidene Fluoride) Membranes with Penetrative Pores for Photothermal Desalination. *Research* **2020**, (2020).

14. Hu, R. *et al.* A Janus evaporator with low tortuosity for long-term solar desalination. *J Mater Chem A Mater* **7**, 15333–15340 (2019).

15. Shao, Y. *et al.* Designing a bioinspired synthetic tree by unidirectional freezing for simultaneous solar steam generation and salt collection. *EcoMat* **2**, (2020).

16. De Yoreo, J. J. Principles of Crystal Nucleation and Growth. *Rev Mineral Geochem* **54**, 57–93 (2003).

17. Aili, A., Ge, Q. & Zhang, T. How Nanostructures Affect Water Droplet Nucleation on Superhydrophobic Surfaces. *J Heat Transfer* **139**, (2017).

18. Lewis, A., Seckler, M., Kramer, H. & van Rosmalen, G. *Industrial Crystallization*. (Cambridge University Press, 2015). doi:10.1017/CBO9781107280427.

19. Desarnaud, J., Derluyn, H., Carmeliet, J., Bonn, D. & Shahidzadeh, N. Metastability Limit for the Nucleation of NaCl Crystals in Confinement. *J Phys Chem Lett* **5**, 890–895 (2014).

20. Morciano, M., Fasano, M., Boriskina, S. V., Chiavazzo, E. & Asinari, P. Solar passive distiller with high productivity and Marangoni effect-driven salt rejection. *Energy Environ Sci* **13**, 3646–3655 (2020).

21. Shahidzadeh-Bonn, N., Rafaï, S., Bonn, D. & Wegdam, G. Salt Crystallization during Evaporation: Impact of Interfacial Properties. *Langmuir* **24**, 8599–8605 (2008).

22. Younes, A., Fahs, M. & Ahmed, S. Solving density driven flow problems with efficient spatial discretizations and higher-order time integration methods. *Adv Water Resour* **32**, 340–352 (2009).

23. Morciano, M., Fasano, M., Boriskina, S. V., Chiavazzo, E. & Asinari, P. Solar passive distiller with high productivity and Marangoni effect-driven salt rejection. *Energy Environ Sci* **13**, 3646–3655 (2020).
