# Supplementary material for: Sustainable biomimetic solar distillation with edge crystallization for passive salt collection and zero brine discharge
Source: Nat Commun. 2024 Jan 29;15:874. doi: 10.1038/s41467-024-45108-2 (PMC10825211; doi:10.1038/s41467-024-45108-2)
Supplement: Supplementary file 3 — Description of Additional Supplementary Files [file 41467_2024_45108_MOESM3_ESM.pdf]

Description of Additional Supplementary Files

File Name: Supplementary Movie 1
Description: Periodic (day & night) operation of the proposed solar vapor generator and crystallizer (SVGC).
